# Efficacy of repair and reconstruction therapy for the treatment of lateral ankle ligament injury: A protocol of systematic review and meta-analysis

**DOI:** 10.1097/MD.0000000000020344

**Published:** 2020-05-29

**Authors:** Zheng-gang Wang, Chao Wu

**Affiliations:** Second Ward of Orthopaedics Department, No. 215 Hospital of Shaanxi Nuclear Industry, Xianyang, Shaanxi, China.

**Keywords:** efficacy, lateral ankle ligament injury, repair and reconstruction therapy

## Abstract

**Background:**

In this study, we will explore the efficacy and safety of repair and reconstruction therapy (RRT) for patients with lateral ankle ligament injury (LALI).

**Methods:**

Searches will be carried out in the Medline, EMBASE, Web of Science, Cochrane Library, PsycINFO, China National Knowledge Infrastructure, along with a comprehensive search of grey literature. All databases will be searched from inception to the March 1, 2020 with no restrictions to language and publication status. Two investigators will independently conduct selection of study, information collection, and risk of bias assessment, respectively. A third investigator will help to solve any different opinions between 2 investigators. RevMan 5.3 software will be utilized for statistical analysis.

**Results:**

This study will assess the efficacy and safety of RRT for patients with LALI through assessing pain intensity, ankle function after ligament injury, time to return to work, time to return to sports, Tegner activity level, quality of life, and adverse events.

**Conclusion:**

This study summarizes latest evidence of RRT for patients with LALI and may provide guidance for clinical practice.

**Study registration number:** INPLASY202040082.

## 1 Introduction

Ankle sprain is a very common sports injury,^[1–4]^ which may cause ligamentous trauma, functional instability, and early degenerative changes.^[5–7]^ The lateral ankle ligament injury (LALI) manifests with pain, swelling, perception of instability, and recurrent ankle sprain,^[8–12]^ which significantly affect quality of life in patients with LALI.^[13–15]^ Despite previous studies have reported that repair and reconstruction therapy (RRT) has been utilized for the management of LALI,^[16–23]^ their results are contradictory. In addition, no published systematic study has investigated the efficacy and safety of RRT for patients with LALI. Thus, the objective of this study is to assess the efficacy and safety of RRT for patients with LALI.

## 2 Methods and analysis

### 2.1 Study registration

This study protocol has been registered on INPLASY202040082. It has been reported in compliance with the guidelines of Preferred Reporting Items for Systematic review and Meta-Analysis Protocols.^[24]^

### 2.2 Eligibility criteria

#### 2.2.1 Study types

This study will include randomized controlled trials (RCTs) assessing the efficacy and safety of RRT for patients with LALI. Any other studies, except RCTs will be removed from this study.

#### 2.2.2 Participant types

We will include any participants who were diagnosed as LALI, regardless their country, background, race, sex, and age.

#### 2.2.3 Intervention types

In the experimental group, the intervention of interest is the use of RRT for the treatment of patients with LALI.

In the control group, comparators can be any treatments, except the RRT.

#### 2.2.4 Outcome types

The primary outcome includes pain intensity. It was measured by any pain scales, such as Visual Analogue Scale. The secondary outcomes comprise of ankle function after ligament injury (measured by Karlsson scoring scale or other scales), time to return to work (days or weeks or months), time to return to sports (days or weeks or months), Tegner activity level, quality of life (measured by Global Quality of Life Scale, or other indexes), and adverse events.

### 2.3 Information sources and search strategy

All information sources will be searched from inception to the March 1, 2020 with no restrictions to language and publication status. The following databases will be utilized for searching Medline, EMBASE, Web of Science, Cochrane Library, PsycINFO, China National Knowledge Infrastructure. Specific search strategy using searching terms has been used for Medline, and has been presented in Table 1. We will also adapt similar detailed search strategies to the other electronic databases.

**Table 1:**
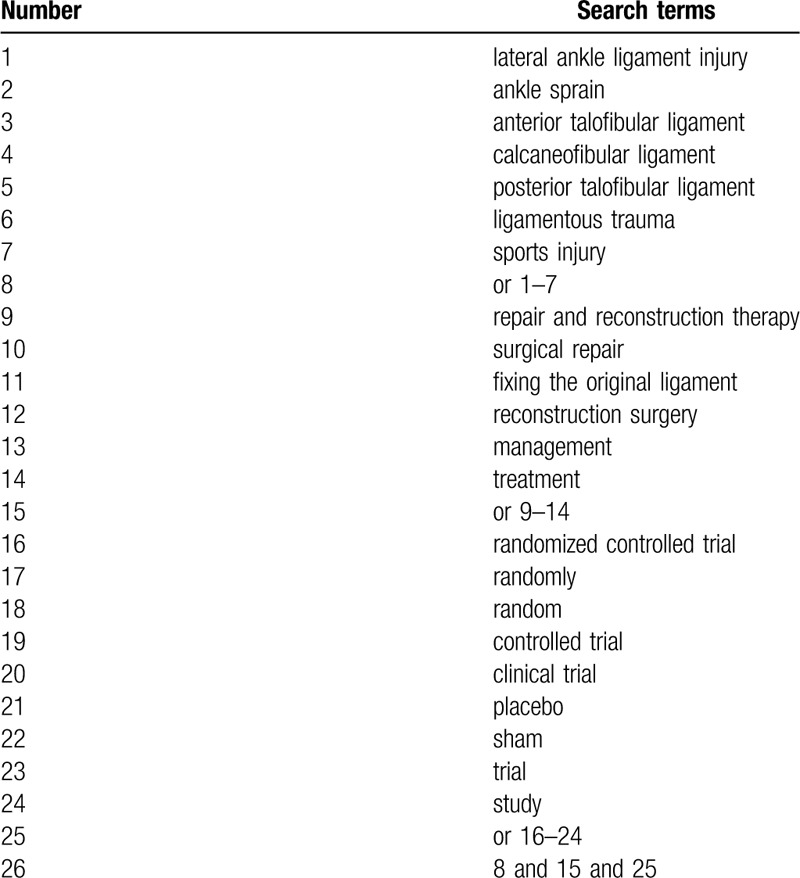
Search strategy utilized for Medline.

In addition, we will also plan to search grey literatures, such as Opengrey, dissertations, conference abstracts, and reference lists of relevant reviews.

### 2.4 Data collection

#### 2.4.1 Study selection

Two investigators will independently undertake study selection in accordance to the previous defined eligible criteria. Titles and abstracts of all citations of searched studies will be checked and studies their content is not relevant to the objectives of this study will be excluded. Then, full papers of remaining records will be read if they meet all inclusion criteria. If there are inconsistent views regarding the study selection between 2 investigators, a third investigator will be asked to make final decision by consultation. The process of study selection will be shown in the flow diagram.

#### 2.4.2 Data collection

Data will be collected by 2 independent investigators using a previous created and standardized data collection sheet. Any discrepancies between 2 investigators will be settled down by a third experienced investigator through discussion. The collected information includes title, first author, year of publication, study design, study setting, patient characteristics, inclusion and exclusion criteria, diagnostic criteria, sample size calculation, details of intervention and controls, outcomes, any expected or unexpected adverse events, and funding information. Whenever there is insufficient information, we will contact original authors to inquire it.

### 2.5 Study quality assessment

Study quality will be assessed by 2 independent investigators with Cochrane Risk of Bias Tool. It is designed for use within all RCTs, and each study will be assessed on 7 aspects according to the guidelines of Cochrane Handbook for Systematic Reviews of Interventions. Using the guidelines, all included studies will be graded as low, unclear or high risk of bias. Any different opinions between 2 investigators will be solved by a third investigator through discussion.

### 2.6 Statistical analysis

We will undertake statistical analysis using RevMan 5.3 software. We will express continuous values as mean difference or standardized mean difference and 95% confidence intervals, and dichotomous values as risk ratio and 95% confidence intervals. A statistical test of heterogeneity will be performed using I^2^ statistic. I^2^ ≤ 50 means low heterogeneity, and a fixed-effects model will be used; while I^2^ > 50% indicates significant heterogeneity, and a random-effects model will be utilized. If there is low heterogeneity among sufficient included studies, we will plan to conduct meta-analysis if it is possible. On the other hand, if there is substantial heterogeneity, we will perform subgroup analysis to check the possible causes for such significant heterogeneity. In addition, we will also present narrative summary according to the different study characteristics, patient characteristics, details of intervention and control, and outcome measurements.

### 2.7 Additional analysis

We will carry outcome subgroup analysis based on the different types of study characteristics, study quality, treatments, and controls.

We will also perform sensitivity analysis to explore the robustness of pooled results by excluding low quality studies.

### 2.8 Reporting bias

We will also plan to perform Funnel plot and Egger regression test to check reporting bias if more than 10 eligible RCTs are included.^[25–26]^

### 2.9 Ethics and dissemination

This study will only collect published records, thus no ethical approval is needed. The results of this study are expected to be submitted for a peer-review publication.

## 3 Discussion

This study will provide an up-to-date investigation of RRT for patients with LALI. Although previous studies have reported to treat LALI effectively using RRT, there are still contradictory conclusions among them and no study address this issue. To our best knowledge, this study will firstly explore the efficacy and safety of RRT for patients with LALI. Its results will provide recommendation for the management of LALI with RRT.

## Author contributions

**Conceptualization:** Zheng-gang Wang, Chao Wu.

**Data curation:** Zheng-gang Wang, Chao Wu.

**Formal analysis:** Zheng-gang Wang, Chao Wu.

**Investigation:** Chao Wu.

**Methodology:** Zheng-gang Wang.

**Project administration:** Chao Wu.

**Resources:** Zheng-gang Wang.

**Software:** Zheng-gang Wang.

**Supervision:** Chao Wu.

**Validation:** Zheng-gang Wang, Chao Wu.

**Visualization:** Zheng-gang Wang, Chao Wu.

**Writing – original draft:** Zheng-gang Wang, Chao Wu.

**Writing – review & editing:** Zheng-gang Wang, Chao Wu.
